# Effects of an LLM-based intelligent adaptive teaching program on clinical risk perception among newly employed nurses: a quasi-experimental study

**DOI:** 10.3389/fmed.2026.1980177

**Published:** 2026-09-30

**Authors:** Xiaobin Qiu, Lihua Shen, Linyan Xie, Liping Wang

**Affiliations:** Nursing Department, Sir Run Run Shaw Hospital, Zhejiang University School of Medicine, Hangzhou, China

**Keywords:** adaptive teaching, adverse events, artificial intelligence, large language model (LLM), newly employed nurses, nursing safety, risk perception

## Abstract

**Objective:**

To evaluate the effectiveness of a large language model (LLM)-based intelligent adaptive teaching program in improving adverse event risk perception among newly employed nurses.

**Methods:**

A prospective quasi-experimental study was conducted among 134 newly employed nurses recruited between July 2023 and July 2025 from a campus of a tertiary Grade A hospital in Zhejiang Province, China. Participants received training using an LLM-based intelligent adaptive teaching program. The Nurses' Risk Perception of Adverse Events Scale (NRPAES), the incidence of nursing adverse events, and training satisfaction were assessed before the intervention and 3 months after training. Differences in these outcomes before and after the intervention were analyzed. Statistical analyses were performed using SPSS version 26.0, with the chi-square test and Wilcoxon signed-rank test used as appropriate.

**Results:**

Following the LLM-based intelligent adaptive training, the adverse event risk perception score among newly employed nurses increased significantly from 14.95 (11.60, 17.29) before training to 16.22 (12.76, 19.00) after training (*Z* = −3.527, *P* < 0.001). The overall incidence of nursing adverse events decreased significantly from 36 events (1.01‰) before training to 28 events (0.58‰) after training (*χ*^2^ = 5.060, *P* = 0.024). Visualization using a Sankey diagram further demonstrated an overall reduction in adverse events after the intervention. In addition, more than 90% of newly employed nurses reported satisfaction with each item of the satisfaction questionnaire for the LLM-based intelligent adaptive teaching program.

**Conclusion:**

The LLM-based intelligent adaptive teaching program provides practical support for the individualized delivery of clinical risk perception training for newly employed nurses. The program may improve nurses' ability to perceive adverse event risks. These findings provide preliminary evidence supporting the integration of LLM-based tools into the design and delivery of nursing education programs.

## 1 Introduction

Risk perception among nursing personnel refers to nurses' attitudes toward, awareness of, and subjective judgments regarding occupational risks (1). As patients' conditions become increasingly complex and diverse, nurses' risk perception has become a critical component of clinical decision-making and patient safety (2). However, according to the World Health Organization (WHO), approximately 1 in 10 patients experience harm while receiving hospital care (3). In response to this challenge, the National Health Commission of China issued the Action Plan for Further Improving Nursing Services (2023–2025) in July 2023, emphasizing the need to ensure nursing quality and safety, reduce the incidence of adverse events, and strengthen nurses' risk perception and risk management capabilities (4). Nurses within the first two years of employment are generally regarded as newly employed nurses (5), and this early stage of clinical practice represents a critical period for developing clinical risk perception. Establishing a strong awareness of patient safety is fundamental to the delivery of safe nursing care. At present, training for newly employed nurses is predominantly based on standardized teaching approaches, which often place insufficient emphasis on individual differences among learners. Such approaches may limit the effective integration of theoretical knowledge with clinical practice and may not adequately prepare newly employed nurses to address complex clinical situations. Consequently, inadequate risk perception remains a common problem during the early stages of professional development (6). Therefore, there is a need to explore teaching approaches that are better adapted to the learning characteristics and needs of newly employed nurses, enhance their engagement in learning, and ultimately improve their clinical risk perception.

The rapid development of large language models (LLMs), particularly generative artificial intelligence represented by DeepSeek, has created new opportunities for nursing education. With capabilities in data processing and analysis, learner profiling, personalized content and learning-path generation, prediction, and decision support, LLMs can provide nursing educators with data-driven early warnings and individualized intervention recommendations (7–9). DeepSeek, in particular, has shown potential advantages in optimizing physician training management, enriching educational resources, and enabling more precise evaluation of training outcomes (10). Although LLM-based technologies have increasingly been explored in physician education and training, reports on their application to the training of newly employed nurses remain limited. Accordingly, the present study developed an LLM-based intelligent adaptive teaching program and applied it to clinical risk perception training for newly employed nurses. The study aimed to evaluate its effects on nurses' risk perception of adverse events, the incidence of nursing adverse events, and training satisfaction, thereby providing evidence for the application of LLM-based tools in the design and delivery of individualized nursing education programs.

## 2 Participants and methods

### 2.1 Participants

This study employed a prospective quasi-experimental design. All newly employed nurses who joined a campus of a tertiary Grade A hospital in Zhejiang Province, China, between July 2023 and July 2025 were recruited as study participants. The inclusion criteria were as follows: (1) completion of a full-time bachelor's degree or higher in nursing; (2) possession of a valid nurse practicing certificate; (3) 0–2 years of work experience; and (4) provision of informed consent and willingness to participate in the study. Nurses who were unable to complete or fully participate in the study because of resignation, sick leave, or other reasons were excluded. The study was approved by the Ethics Committee of Sir Run Run Shaw Hospital, Zhejiang University School of Medicine (Approval No. 2025-Research-059). No known major medical safety interventions or changes to the training system occurred during the study period. However, there were fluctuations in the number of inpatients, primarily related to the Spring Festival and seasonal factors.

### 2.2 Training methods

#### 2.2.1 Establishment of the LLM-based intelligent adaptive clinical risk perception training team for newly employed nurses

A multidisciplinary training team was established to develop and implement the LLM-based intelligent adaptive clinical risk perception training program for newly employed nurses. The team comprised 12 members, including one hospital-wide quality and safety administrator, one deputy director of the Nursing Department, one departmental head nurse, three ward head nurses, four nurse educators, and one instructor holding an instructor-level certificate in Artificial Intelligence Generated Content (AIGC) issued by the Ministry of Industry and Information Technology of China.

#### 2.2.2 Identification of teaching and learning problems

##### 2.2.2.1 Baseline knowledge and training background of newly employed nurses

Before implementation of the LLM-based intelligent adaptive teaching program, newly employed nurses had completed the hospital's routine training program appropriate to their respective professional levels. The institutional training program was structured around eight core nursing competencies: assessment and intervention, communication, critical thinking, interpersonal relationships, management and leadership, teaching, research and evidence-based practice, and knowledge integration (11). The components related to clinical risk perception were organized according to the following stages. During the pre-employment training stage (August–December of the year of employment), training related to system and process risks included standardized communication, nurse–physician communication, interpersonal communication skills, and occupational protection for nurses. Training related to nursing procedural risks included standardized medication verification and administration procedures, correct specimen collection, personal protective practices for infection prevention and control, establishment and maintenance of intravenous access, and medication administration and management. Training related to patient-care risks included emergency response training and emergency drills organized by individual clinical units. During the first stage (Year 1), training related to system and process risks included interpretation of the core values of nursing, health education and its implementation, general nursing training, humanistic care, and reflective journal writing. Training related to nursing procedural risks included two-person emergency response assessments, hospital-wide nursing skills assessments, and training on medications commonly used within each specialty. Training related to patient-care risks included assessment of knowledge regarding hospital policies and emergency response capabilities. During the second stage (Year 2), training related to system and process risks included clinical rotation and courses on nonviolent communication. Training related to nursing procedural risks included specialty-specific nursing skills assessments, whereas training related to patient-care risks included preparation and presentation of case analyses.

##### 2.2.2.2 Assessment of clinical risk perception among newly employed nurses

Before the training, clinical risk perception among newly employed nurses was assessed using a questionnaire. The results showed that the system and process risk perception dimension had the highest score, at 15.67 (12.11, 18.43), followed by nursing procedural risk perception, at 14.88 (11.50, 17.44). The patient-care risk perception dimension had the lowest score, at 14.42 (11.11, 16.89). These scores were lower than the average levels reported for nurses in comparable hospitals in China (12, 13).

##### 2.2.2.3 Analysis of clinical adverse event cases

Cases of adverse events involving newly employed nurses during clinical practice in the first quarter of 2025 were extracted from the hospital's adverse event reporting system. Information collected for each case included a 5W2H-based description of the event, event severity, event classification, and the principal methods and measures proposed to prevent recurrence. The adverse event data were subsequently analyzed using MicroWordCloud to identify frequently occurring terms and generate a keyword cloud. The analysis identified four major categories of keywords: policies and procedures, personnel management, communication, and education.

#### 2.2.3 Development of the LLM-based intelligent adaptive teaching program

The teaching program was developed using an LLM-based intelligent adaptive approach. First, a standardized instructional prompt was developed. Drawing on relevant domestic and international literature (9, 14), the research team used KIMI to assist with prompt drafting and repeatedly refined the prompt through group discussions and iterative testing. A structured prompt format was ultimately established to ensure clear language, standardized terminology, and controllable output. The prompt consisted of three components: role definition, task objectives, and output requirements. The specific prompt was as follows: You are an expert with extensive experience in nursing education. Your task is to design a teaching program on risk perception training for a nursing team, with the aim of improving newly employed nurses' ability to perceive clinical risks, thereby improving patient outcomes and the quality of nursing care. Please carefully review the four uploaded files. The first file contains the results of a clinical risk perception survey among newly employed nurses; the second summarizes adverse events involving newly employed nurses; the third contains the training content that newly employed nurses have already completed; and the fourth provides an example of a high-quality teaching program. Based on the 4F principle—Fatal, Frequent, Fundamental, and Fixed—develop a corresponding teaching program for newly employed nurses. The program should include teaching objectives, teaching content, teaching methods, teaching tools, teaching evaluation, and time allocation. Content that has already been covered in previous training for newly employed nurses should be excluded. The teaching program should be based on the latest evidence-based medical evidence and nursing guidelines, reflect actual clinical practice needs, and be appropriate for the professional level and learning characteristics of newly employed nurses, with particular emphasis on practical skills and case-based analysis. The final teaching program should be generated in Microsoft Word format.

Second, the instructional prompts and source materials were entered into the LLM platforms for autonomous content generation. To ensure the reliability of the input information, two researchers independently verified all materials before submission. After confirmation, a nursing expert who held an AIGC certification entered the materials into DeepSeek, Doubao, and KIMI, respectively. DeepSeek was operated in its deep-thinking mode. Each platform independently analyzed the materials and generated a clinical risk perception training program for newly employed nurses. The generated programs were downloaded and saved in Microsoft Word format. Two researchers then independently collected, consolidated, and cross-checked the generated teaching programs to minimize subjective bias and improve the reliability of the resulting content. Following synthesis and refinement of the LLM-generated outputs, three major teaching modules were identified: Module 1, Systems, Processes, and Institutional Standards, focusing on institutional procedures and clinical documentation; Module 2, Prevention and Control of Nursing Procedural Risks, focusing on complex scenarios involving specimen collection; and Module 3, Patient-Care Risks and Humanistic Nursing, focusing primarily on communication.

Third, the teaching programs were evaluated and finalized. With assistance from KIMI, a standardized evaluation prompt was developed using the same three-component structure. The prompt was as follows: You are an expert with extensive experience in nursing education and are responsible for evaluating clinical nursing lesson plans developed by clinical nurse educators. You have expertise in nursing, education, and curriculum design, as well as an in-depth understanding of the design and evaluation of high-quality nursing lesson plans. You should be capable of evaluating lesson plans, assessing instructional objectives, analyzing course content, evaluating teaching methods, and providing constructive feedback. Please comprehensively evaluate the scientific rigor, practical applicability, achievement of teaching objectives, and effectiveness of teaching methods of the lesson plan, and provide professional comments and recommendations for improvement. The evaluation should be objective and impartial to ensure accuracy and constructiveness, while adhering to educational ethics and confidentiality requirements. The evaluation report should include an overall assessment, strengths, limitations, recommendations, and an overall conclusion. The evaluation tasks are as follows: (1) review and understand the teaching objectives, course content, teaching methods, and assessment methods; (2) evaluate the scientific rigor and practical applicability of the lesson plan and determine whether its content is consistent with nursing education standards and clinical practice requirements; (3) assess whether the teaching objectives are clearly defined and aligned with Bloom's taxonomy of educational objectives; (4) evaluate whether the course content is comprehensive, accurate, and logically organized; (5) assess whether the teaching methods are diverse, effective, and appropriate for learners' learning characteristics; (6) determine whether the assessment methods are appropriate, fair, and capable of effectively monitoring learners' progress; (7) identify strengths of the lesson plan, such as innovative teaching approaches, clinically relevant content, or clearly defined learning objectives; and (8) identify limitations, including restricted content coverage, insufficient diversity of teaching methods, or weaknesses in the assessment strategy. The standardized evaluation prompt together with the generated lesson plans was subsequently uploaded to Yuanbao AI for evaluation and revision. Based on the AI-generated evaluation results and subsequent refinement by the research team, the teaching programs for the three thematic modules were finalized, as shown in Table 1.

**Table 1:** Teaching program for clinical risk perception training among newly employed nurses.

Module	Teaching objectives	Teaching content	Teaching methods	Teaching tools	Teaching evaluation	Duration (45 min/session)
Module 1: System and Process Risk Perception	Knowledge objective: Participants will be able to summarize key considerations in nursing documentation. Skill objective: Participants will be able to correctly demonstrate standardized nursing documentation. Affective objective: Participants will recognize the importance of standardized nursing documentation	High-risk points in systems and processes: 1. Standardization of nursing documentation, including special care records and resuscitation records	1. Case-based learning (CBL) 2. Problem-based learning (PBL)	1. PowerPoint courseware 2. Error cases	1. Group discussion 2. Outcome presentation	1 session
Module 2: Nursing Procedural Risk Perception	Knowledge objective: Participants will be able to identify problems in standardized specimen verification and specimen handling. Skill objective: Participants will be able to correctly demonstrate standardized verification and specimen handling procedures. Affective objective: Participants will recognize the risks associated with nonstandard specimen verification and handling	High-risk points in standardized procedures: 1. Medication verification procedures, including post-interruption rechecking 2. Specimen collection in complex clinical scenarios 3. Specimen handling, including special considerations within the hospital	1. Procedural demonstration 2. Scenario-based simulation 3. Return demonstration	1. Operational scenario scripts 2. Procedure flowcharts/videos	1. Procedural performance checklist 2. Role-play observation form	2 session
Module 3: Patient-Care Risk Perception	Knowledge objective: Participants will be able to identify key elements of effective communication. Skill objective: Participants will be able to correctly demonstrate standardized communication procedures. Affective objective: Participants will demonstrate the ability to manage communication-related crises in nursing care	Communication-related risk points: 1. Communication with general ward patients 2. Communication with emergency department patients 3. Communication with intensive care unit patients	1. Case-based learning (CBL) 2. Scenario-based simulation	1. Patient-care risk scenario scripts	1. Case analysis presentation and quality assessment	3 session

### 2.3 Outcome measures

#### 2.3.1 Clinical risk perception among newly employed nurses

Clinical risk perception was assessed using the Nurses' Risk Perception of Adverse Events Scale, developed by Mao Qiuyun (13). The scale consists of 20 items across three dimensions: patient-care risk, system and process risk, and nursing procedural risk. Each item is evaluated from two perspectives: the likelihood of an adverse event occurring and the severity of its consequences. The likelihood of occurrence is rated on a 6-point Likert scale, ranging from 1 (“extremely unlikely”) to 6 (“very likely”). The severity of consequences is rated on a 5-point Likert scale, ranging from 1 (“not serious at all”) to 5 (“very serious”). The score for each item is calculated by multiplying the likelihood score by the severity score, with higher scores indicating stronger perception of adverse event risk among clinical nurses. The scale has demonstrated good reliability, with a Cronbach's *α* coefficient of 0.939.

#### 2.3.2 Incidence of nursing safety adverse events

The incidence of nursing adverse events during the 3 months before and after the intervention was compared. The adverse events assessed included medication errors, falls, unplanned extubation, and pressure injuries (15). Nursing adverse events were classified into four levels: Level I sentinel events, Level II adverse-outcome events, Level III no-harm events, and Level IV near-miss events. Data on nursing safety adverse events were extracted by the researchers from the hospital's adverse event reporting system and subsequently reviewed and confirmed by the Nursing Quality and Safety Management Committee. The incidence of nursing adverse events was calculated as follows: Incidence of adverse events (‰) = number of adverse events/total number of hospitalized patients during the study period × 1,000‰. The number of adverse events refers to those associated with newly employed nurses, with the denominator being the total number of inpatients during the study period. This metric reflects the incidence of adverse events associated with newly employed nurses among all inpatients, it is not an incidence rate calculated based on the number of newly employed nurses. A Sankey diagram (16), a specific type of flow diagram, was used to visualize the flow and distribution of different categories of adverse events across interconnected nodes. This visualization facilitates the identification and presentation of underlying patterns and supports interpretation, analysis, and decision-making. In the present study, Sankey diagrams were used to compare overall changes in the flow of different types of adverse events before and after training, thereby providing an intuitive visualization of the effects of the training intervention.

#### 2.3.3 Training satisfaction

After completion of the training, participants completed a self-administered satisfaction questionnaire developed by the hospital. The questionnaire comprised five dimensions: teaching content, achievement of course objectives, teaching methods, duration of instruction, and instructors' teaching skills. Each item was rated on a 5-point Likert scale, with 1 indicating “very dissatisfied,” 2 “dissatisfied,” 3 “neutral,” 4 “satisfied,” and 5 “very satisfied.” Higher scores indicated greater satisfaction with the training program.

### 2.4 Statistical analysis

Statistical analyses were performed using SPSS version 26.0. Categorical variables were summarized as frequencies and percentages, and comparisons were performed using the chi-square test. Continuous variables were assessed for normality. Variables that were not normally distributed were expressed as the median and interquartile range [M (P25, P75)] and compared using the Wilcoxon signed-rank test. A two-sided *P* value < 0.05 was considered statistically significant.

## 3 Results

### 3.1 Participant characteristics

A total of 134 newly employed nurses were included in the study, comprising 8 men and 126 women. The mean age of the participants was 24.17 ± 1.41 years (Table 2). The flowchart of Researcher Participation is as follows (Figure 1).

**Table 2:** General characteristics of newly employed nurses (*n* = 134).

Variable	Category	n	Percentage (%)
Sex	Male	8	6.0
	Female	126	94.0
Educational level	Bachelor's degree	128	95.5
	Master's degree	6	4.5
Marital status	Unmarried	130	97.0
	Married	4	3.0
Place of residence	Urban	66	49.3
	Rural	68	50.7
Only-child status	Yes	40	29.9
	No	94	70.1
Ethnicity	Han	129	96.3
	Other ethnic groups	5	3.7
Years of work experience	0 years	55	41.0
	1 years	58	43.3
	2 years	21	15.7
Professional title	Nurse	78	58.2
	Junior nurse*	56	41.8
Number of night shifts per month	≤5	122	91.0
	6–10	12	9.0
Availability of comprehensive patient safety training in the department	Yes	134	100
	No	0	0
Previous involvement in nursing adverse events	Yes	68	50.7
	No	66	49.3
Self-rated communication ability	Very good	3	2.2
	Good	82	61.2
	Average	49	36.6
Self-rated work-related stress	Very high	4	3.0
	Relatively high	55	41.0
	Moderate	70	52.2
	Relatively low	5	3.7
Self-perceived burnout	None	34	25.4
	Mild	80	59.7
	Moderate	16	11.9
	Severe	4	3.0

**Figure 1:**
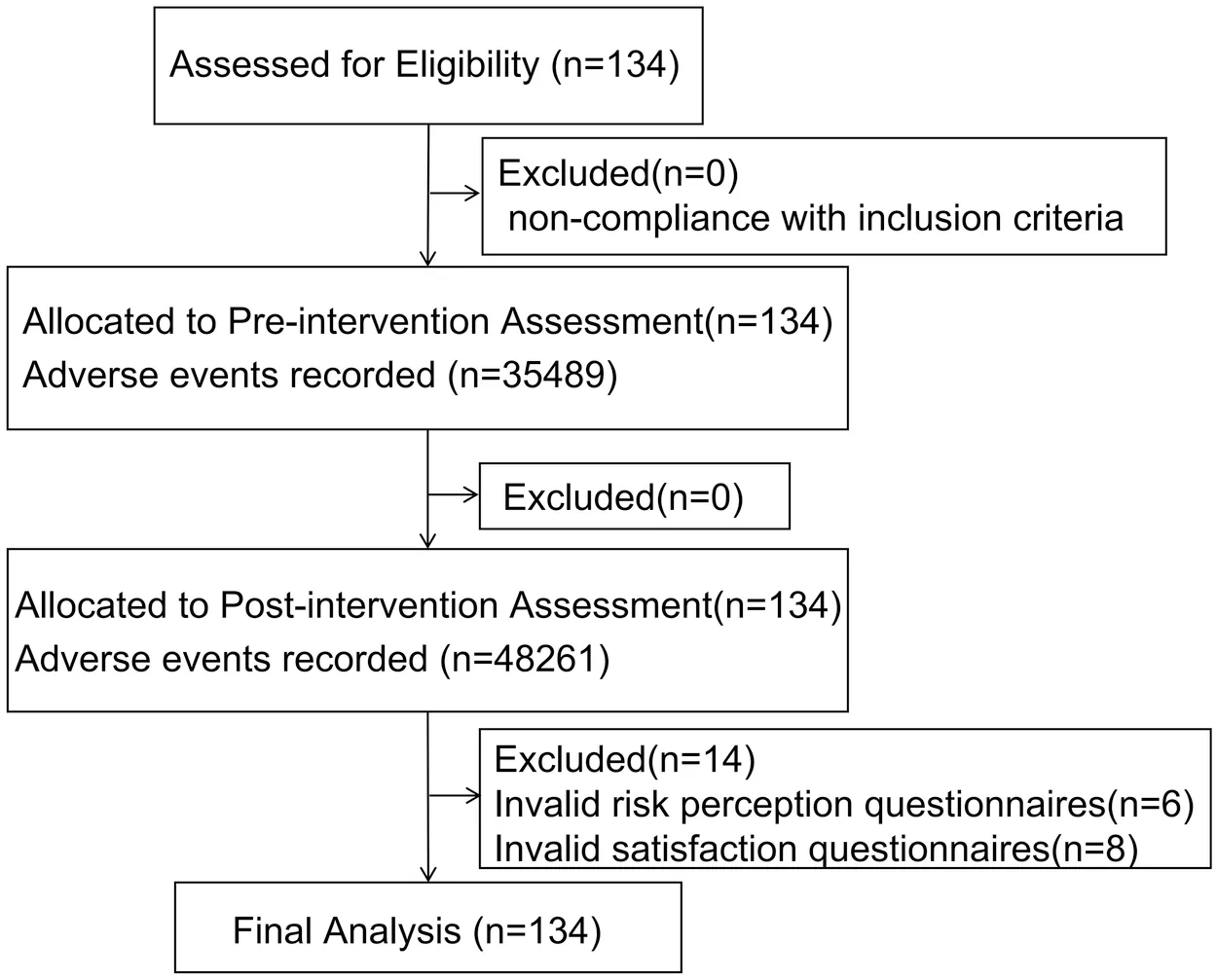
Flowchart of researcher participation.

### 3.2 Self-assessment of clinical risk perception before and after training

A total of 134 questionnaires were distributed, of which 6 were excluded as invalid. Consequently, 128 valid questionnaires were included in the analysis, yielding a valid response rate of 95.52%. After training, the overall score for adverse event risk perception among newly employed nurses was significantly higher than that before training (*P* < 0.05). Scores for the nursing procedural risk and patient-care risk dimensions also increased significantly after training (*P* < 0.05). However, no statistically significant difference was observed in the system and process risk dimension before vs. after training (Table 3).

**Table 3:** Self-assessment of clinical risk perception before and after training.

Category	Group	Score	*Z*	*P*
Adverse event risk perception	Before intervention	14.95 (11.60,17.29)	−3.527	<0.001
	After intervention	16.22 (12.76,19.00)		
Nursing procedural risk	Before intervention	14.88 (11.50,17.44)	−2.305	0.021
	After intervention	15.93 (12.00,19.62)		
Patient-care risk	Before intervention	14.42 (11.11,16.89)	−4.697	<0.001
	After intervention	16.25 (12.94,18.81)		
System and process risk	Before intervention	15.67 (12.11,18.43)	−1.597	0.11
	After intervention	16.35 (13.68,19.50)		

### 3.3 Incidence of nursing adverse events before and after training

After training, a total of 28 nursing adverse events were reported among newly employed nurses, corresponding to an incidence of 0.58‰ (28/48,261). In comparison, before training, 36 adverse events were reported, corresponding to an incidence of 1.01‰ (36/35,489). The overall incidence of nursing adverse events among newly employed nurses was significantly lower after training than before training (*P* < 0.05) (Table 4). A Sankey diagram was used to visualize changes in the composition and flow of nursing adverse events before and after the intervention (Figure 2). In the diagram, the width of each flow line represents the proportion of a given adverse event category, and the value labeled on each flow line indicates the corresponding proportion. Before the intervention, unplanned extubation accounted for the highest proportion of adverse events (0.48‰), followed by medication errors (0.25‰) and falls (0.20‰), whereas pressure injuries accounted for the lowest proportion (0.09‰). After the intervention, the proportion of unplanned extubation decreased to 0.25‰, representing the most marked reduction. The proportion of medication errors fell from 0.25‰ to 0.10‰, representing a relatively significant decrease. The proportion of falls decreased slightly from 0.20‰ to 0.15‰. The proportion of pressure injuries decreased marginally from 0.09‰ to 0.08‰.

**Table 4:** Comparison of the incidence of nursing adverse events before and after the intervention (‰).

Group	Number of cases, n	Nursing adverse events				
		Overall	Medication errors	Falls	Unplanned extubation	Pressure injuries
Before intervention	35,489	36 (1.01)	9 (0.25)	7 (0.20)	17 (0.48)	3 (0.09)
After intervention	48,261	28 (0.58)	5 (0.10)	7 (0.15)	12 (0.25)	4 (0.08)
*χ* ^2^		5.060	2.753	0.333	3.136	0.001
*P*		0.024	0.097	0.564	0.077	0.979

**Figure 2:**
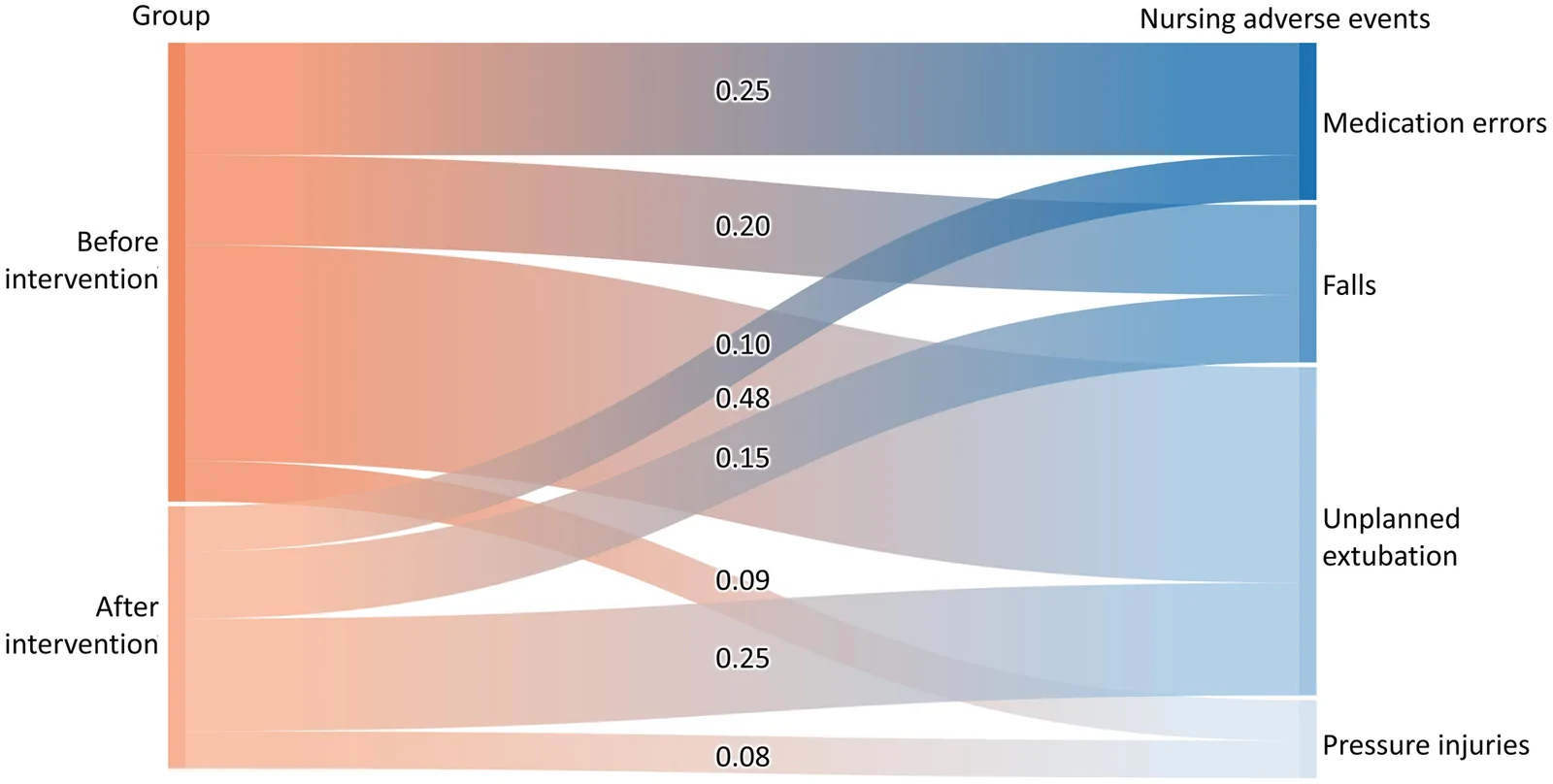
Sankey diagram showing the distribution of nursing adverse events before and after the intervention.

### 3.4 Satisfaction with the LLM-based intelligent adaptive teaching program among newly employed nurses

A total of 134 questionnaires were distributed, of which 8 were excluded as invalid. Regarding teaching content, 93.65% (118/126) of participants reported being “very satisfied,” while the remainder reported being “satisfied,” indicating a high level of satisfaction with the relevance and value of the course content. For achievement of course objectives, 94.44% (119/126) of participants reported being “very satisfied,” while the remainder reported being “satisfied,” suggesting that the training largely met their expected learning objectives. Regarding teaching methods, 94.44% (119/126) of participants reported being “very satisfied,” and the remainder reported being “satisfied,” indicating a high level of acceptance of the instructional approaches used. For training duration, 79.37% (100/126) of participants reported being “very satisfied,” 15.87% (20/126) reported being “satisfied,” and 4.76% (6/126) reported a “neutral” level of satisfaction. Compared with the other dimensions, satisfaction with training duration was relatively lower, suggesting room for further optimization. Regarding instructors' teaching skills, 93.65% (118/126) of participants reported being “very satisfied,” while the remainder reported being “satisfied,” indicating a high level of satisfaction with the instructors' teaching performance.

## 4 Discussion

### 4.1 The LLM-based intelligent adaptive teaching program improved clinical risk perception and professional competence among newly employed nurses

The results showed that adverse event risk perception among newly employed nurses improved significantly after training compared with that before training (*P* < 0.05). This finding suggests that the LLM-based intelligent adaptive teaching program may help enhance newly employed nurses' ability to perceive risks associated with adverse events. Two main factors may account for this improvement. First, the present study used a clinical risk perception questionnaire to identify difficulties encountered by newly employed nurses in relation to system and process risks, nursing procedural risks, and patient-care risks. Based on these identified needs, the training content was tailored to better align educational provision with actual clinical demands. In addition, MicroWordCloud analysis was used to summarize patterns in clinical adverse events. This data-driven approach enabled the research team to adjust teaching priorities according to actual adverse event data and to develop a training program that more closely matched the clinical risk profile of newly employed nurses. Second, previous studies have shown that targeted case-based analysis (17) and simulation-based training (18) can facilitate the identification and management of potential clinical risks among newly employed nurses. In the present study, real-world case analysis was combined with simulated interactive training. This approach may have strengthened participants' recognition of clinical risks and improved their judgment and decision-making when managing complex clinical situations, thereby contributing to enhanced professional competence.

However, no statistically significant improvement was observed in the system and process risk dimension after training. One possible explanation is that some system- and process-related risks, particularly inaccuracies in nursing documentation, may not be sufficiently captured by self-reported scale-based assessments. High-quality nursing documentation requires compliance with institutional standards, accuracy of written records, and consistency with actual clinical events. Therefore, objective assessment based on sampling audits and cross-validation may provide a more reliable measure of improvement in this domain. Future studies should consider incorporating standardized audits of nursing documentation conducted by trained instructors, together with clearly defined scoring criteria and sampling procedures. Such objective assessments may provide a more comprehensive and accurate evaluation of changes in documentation performance and the effectiveness of training among newly employed nurses.

### 4.2 The LLM-based intelligent adaptive teaching program reduced the incidence of nursing adverse events and promoted patient safety

Nursing quality and safety adverse events are important indicators for risk surveillance and early warning in hospital risk management (19). Previous studies have shown that newly employed nurses recognize the importance of clinical safety and that adherence to high standards of clinical safety practice may improve professional skills and reduce the occurrence of adverse events (20). In the present study, visualization using a Sankey diagram showed that the proportions of unplanned extubation and fall events decreased after the intervention, suggesting that the teaching program may have had beneficial effects on catheter safety management and fall prevention. Overall, the incidence of nursing adverse events was significantly lower after the intervention than before the intervention. One possible explanation is that the program incorporated instructional strategies such as procedural demonstrations and scenario-based enactment to review and reconstruct adverse event situations. These approaches may have enabled newly employed nurses to more directly recognize the potential consequences of adverse events, strengthen their awareness of clinical safety and risk prevention, and promote more standardized nursing practices. This finding is consistent with previous evidence indicating that standardized bedside nursing processes can improve nurses' safety awareness and safety-related behaviors (21). In addition, the LLM-based adaptive approach enabled the training content to be tailored more closely to the characteristics of adverse events commonly encountered by newly employed nurses. By identifying frequent deficiencies in clinical procedures, the program helped participants recognize potential gaps in their individual workflows. Subsequent return demonstrations reinforced standardized procedures and may have facilitated the consolidation of safe nursing behaviors and improved adherence to clinical safety practices, thereby contributing to an overall reduction in the risk of nursing adverse events and enhanced patient safety.

However, the Sankey diagram also showed that the proportion of medication errors decreased significantly, yet this finding lacked statistical significance, suggesting that the current comprehensive intervention may have provided insufficiently targeted training for medication safety. Although the intervention was associated with a significant reduction in the overall incidence of nursing adverse events, analyses of individual event categories did not demonstrate statistically significant differences. This may partly be attributable to the small number of events within each category after the overall adverse events were subdivided into falls, pressure injuries, medication errors, and other event types. The resulting small expected frequencies may have reduced statistical power and limited the ability to detect potentially meaningful differences within individual categories. Furthermore, the teaching program was designed as a multidimensional and systematic quality-improvement strategy aimed primarily at strengthening overall nursing safety rather than addressing a single specific risk. Accordingly, its effects may be more readily reflected in the aggregate incidence of nursing adverse events than in individual event categories. Taken together, these findings suggest that the LLM-based intelligent adaptive teaching program has practical value for improving nursing safety and supporting patient safety, while more targeted interventions may be required to address specific risks such as medication errors.

### 4.3 Newly employed nurses reported high satisfaction with the LLM-based intelligent adaptive teaching program

More than 90% of participants reported being satisfied or very satisfied with each item of the training satisfaction questionnaire, indicating a high level of acceptance of the LLM-based intelligent adaptive teaching program among newly employed nurses. From the perspective of instructional design and implementation, this high satisfaction may be attributable to the targeted design of the training content, the structured organization of teaching activities, and the use of diversified instructional methods. The training content was developed on the basis of weaknesses identified among newly employed nurses themselves, which may have enhanced their interest and active engagement in learning. Linking clinical cases with relevant knowledge points also facilitated the construction of a more systematic framework for clinical risk perception. Previous studies have suggested that LLM-based educational tools can improve the adaptability of teaching programs to learners' needs and may enhance learning attitudes and self-directed learning (22). During program development, the LLM organized the identified risk-related knowledge according to the 4F principle and structured it in a manner aligned with the cognitive characteristics of newly employed nurses, thereby facilitating the development of individualized learning frameworks, highlighting key learning priorities, and potentially improving teaching efficiency (23, 24). Compared with traditional educator-centered teaching, the present program placed greater emphasis on learner participation. Newly employed nurses were continuously encouraged to engage in, experience, and share the learning process, which may have enhanced their initiative, autonomy, and learning interest. This learner-centered and adaptively structured approach may therefore have contributed to the high level of satisfaction observed in the present study. In addition, an efficient and targeted teaching approach may help reduce unnecessary learning burden and provide a more focused learning experience, supporting the potential application of LLM-assisted instructional design in nursing education.

Nevertheless, satisfaction with the duration of training was relatively lower than that for the other dimensions, with only 79.37% of participants reporting that they were “very satisfied.” This may be because the practical training sessions combined real-world case analysis with simulation-based interaction and therefore required sufficient time for scenario enactment and discussion. Future training programs could optimize time allocation by summarizing core content using concise memory aids, introducing time-limited questioning during interactive sessions, and reducing unnecessary waiting periods while maintaining participant engagement. Virtual reality technology could also be explored in future risk perception training to provide a more immersive and realistic learning environment.

## 5 Conclusion

This study targeted weaknesses in clinical risk perception among newly employed nurses and developed an LLM-based intelligent adaptive teaching program accordingly. The findings showed that the program was associated with improved clinical risk perception, a lower overall incidence of nursing adverse events, and a high level of training satisfaction among newly employed nurses. LLMs have considerable potential for supporting the development of adaptive teaching programs; however, limitations remain in terms of rigor, professional accuracy, and the reliability of generated content. Therefore, teaching programs generated with LLM assistance should undergo systematic professional evaluation and review before implementation. Several limitations of this study should be acknowledged. First, this study employed a single-group pre-post design without a parallel control group; therefore, the potential effects of confounding factors, such as the accumulation of clinical experience among newly employed nurses, natural maturation, and repeated testing, could not be fully excluded. Second, participants were followed for only three months after the training, which limited our ability to determine the long-term sustainability of the educational effects or to assess whether these effects translated into sustained improvements in clinical practice and patient safety outcomes. There were certain differences in the total number of hospitalized patients between the two periods. The total number of patients after the intervention increased by approximately 36% compared with that before the intervention, which may be related to healthcare-seeking behaviors around the Spring Festival, the scheduling of elective surgeries, and seasonal variations in disease prevalence. The patient populations may not be fully comparable. Although the chi-square test was applied, residual confounding may still exist. Third, clinical risk perception was assessed primarily using self-report measures, which may have introduced response and reporting biases. Future studies should adopt more rigorous designs, preferably randomized controlled trials with conventional teaching as a comparator, extend the follow-up period, and incorporate scenario-based simulations, objective assessments of clinical behavior, and patient safety-related outcomes. Such approaches would enable a more comprehensive evaluation of the effectiveness, durability, and clinical relevance of large language model-based intelligent adaptive teaching programs.

## Data Availability

The original contributions presented in the study are included in the article/Supplementary Material, further inquiries can be directed to the corresponding author/s.
